# HIV-Infected Spleens Present Altered Follicular Helper T Cell (Tfh) Subsets and Skewed B Cell Maturation

**DOI:** 10.1371/journal.pone.0140978

**Published:** 2015-10-26

**Authors:** Lucie Colineau, Angeline Rouers, Takuya Yamamoto, Yin Xu, Alejandra Urrutia, Hang-Phuong Pham, Sylvain Cardinaud, Assia Samri, Karim Dorgham, Pierre-Grégoire Coulon, Rémi Cheynier, Anne Hosmalin, Eric Oksenhendler, Adrien Six, Anthony D. Kelleher, John Zaunders, Richard A. Koup, Brigitte Autran, Arnaud Moris, Stéphanie Graff-Dubois

**Affiliations:** 1 Sorbonne Universités, UPMC Université Paris 06, Center for Immunology and Microbial Infections—CIMI-Paris, Paris, France; 2 INSERM, U1135, Center for Immunology and Microbial Infections—CIMI-Paris, Paris, France; 3 CNRS, ERL 8255, Center for Immunology and Microbial Infections—CIMI-Paris, Paris, France; 4 Immunology Laboratory, Vaccine research center, National Institute of Allergy and Infectious Diseases (NIAID), NIH, Bethesda, Maryland, United States of America; 5 The Kirby Institute for Infection and Immunity in Society, University of New South Wales, Sydney, Australia; 6 Sorbonne Universités UPMC Université Paris 06, UMRS 959, Immunology-Immunopathology-Immunotherapy (I3), Paris, France; 7 INSERM, UMRS 959, Immunology-Immunopathology-Immunotherapy (I3), Paris, France; 8 CNRS, FRE3632, Immunology-Immunopathology-Immunotherapy (I3), Paris, France; 9 INSERM, U1016, Institut Cochin, Paris, France; 10 CNRS, UMR8104, Paris, France; 11 Université Paris Descartes, Sorbonne Paris Cité, Paris, France; 12 AP-HP, Hôpital Cochin, Paris, France; 13 Université Paris Diderot, Assistance Publique-Hôpitaux de Paris, Département d’Immunologie Clinique, Hôpital Saint-Louis, Paris, France; 14 St. Vincent's Centre for Applied Medical Research, St. Vincent's Hospital, Sydney, Australia; 15 AP-HP, Hôpital Pitié-Salpêtière, Department of Immunology, Paris, France; Jackson Laboratory, UNITED STATES

## Abstract

Follicular helper T (Tfh) cells within secondary lymphoid organs control multiple steps of B cell maturation and antibody (Ab) production. HIV-1 infection is associated with an altered B cell differentiation and Tfh isolated from lymph nodes of HIV-infected (HIV^+^) individuals provide inadequate B cell help *in vitro*. However, the mechanisms underlying this impairment of Tfh function are not fully defined. Using a unique collection of splenocytes, we compared the frequency, phenotype and transcriptome of Tfh subsets in spleens from HIV negative (HIV^-^) and HIV^+^ subjects. We observed an increase of CXCR5^+^PD-1^high^CD57^-^Tfh and germinal center (GC) CD57+ Tfh in HIV^+^ spleens. Both subsets showed a reduced mRNA expression of the transcription factor STAT-3, co-stimulatory, regulatory and signal transduction molecules as compared to HIV^-^ spleens. Similarly, Foxp3 expressing follicular regulatory T (Tfr) cells were increased, suggesting sustained GC reactions in chronically HIV^+^ spleens. As a consequence, GC B cell populations were expanded, however, complete maturation into memory B cells was reduced in HIV^+^ spleens where we evidenced a compromised production of B cell-activating cytokines such as IL-4 and IL-10. Collectively our data indicate that, although Tfh proliferation and GC reactions seem to be ongoing in HIV-infected spleens, Tfh “differentiation” and expression of costimulatory molecules is skewed with a profound effect on B cell maturation.

## Introduction

T follicular helper cells (Tfh) are a specialized subset of CD4 T cells that support the multiple steps of B cell maturation and differentiation into long-lived plasma cells. Tfh cells are characterized by the expression of the chemokine (C-X-C motif) receptor (CXCR) 5 that allows their migration towards the chemokine (C-X-C motif) ligand (CXCL) 13, a chemokine strongly expressed in the follicles of the spleen, lymph nodes, and Peyer’s Patches. In these follicles, Tfh cells provide cognate help to B cells, promoting germinal center (GC) B cell responses and long-term protective humoral immunity. Tfh cells are characterized by a very high expression of co-stimulatory molecules involved in B help such as Inducible T-cell Costimulator (ICOS), OX40 and CD40 ligand (CD40L) [1]. They also express high levels of Programmed Death-1 (PD-1). One functional feature of Tfh cells is their high capacity to secrete Interleukin-21 (IL-21) and IL-4, which are necessary for GC formation and B cell differentiation into long-lived plasma cells, respectively [2,3]. In addition to the lineage-specific transcriptional regulator B cell lymphoma-6 (Bcl6), transcription factor achaete-scute homologue 2 (ASCL2) is selectively upregulated in Tfh cells and initiates their development [4]. Tfh cell differentiation may be driven by ICOS [5] and CD28 [6] signals during the priming by dendritic cells (DCs) of CD4 T cells into Tfh and during Tfh expansion, particularly in response to infection. After priming, membrane expression of chemokine receptors CCR7 is down-regulated while CXCR5 is up-regulated thus allowing Tfh cells to migrate towards the follicle. The first antigen specific interaction between Tfh and B cells takes place at the T/B border in secondary lymphoid organs. The second interaction occurs within the germinal center between GCTfh and GC B cells. CD40L/CD40, OX40/OX40L, Signaling Lymphocyte Activation Molecule (SLAM) family members and adhesion molecules that strengthen GCTfh / GC B cell interaction are required for full B cell maturation. These interactions activate B cell somatic hyper-mutation of Ig variable regions and selection of high-affinity B cells. Of note, GCTfh cell maintenance requires sustained antigenic stimulation by GC B cells. Thus Tfh and GC B cell numbers are closely interrelated [7].

Recently, T follicular regulatory cells (Tfr) have been described as the regulatory counterpart of the Tfh population [8–11]. Tfr most probably derive from natural T regulatory cells that up-regulate Bcl6 and migrate into follicles after up-regulation of CXCR5 and PD-1 [9]. Hence, Tfr cells share features with both conventional Treg cells and Tfh cells. Similarly to Tregs, they express the typical markers Forkhead Box P3 (Foxp3) and CTLA-4. Tfr also produce high amounts of IL-10 and control the GC reaction to prevent the emergence of autoantibodies [9–12].

In lymph nodes, Tfh cells expand during the chronic phase of HIV infection [13,14]. The expansion of Tfh cells is highly associated with the viremia, along with increased GC B cell and plasma cell populations [14]. Similarly, in the non-human primate model of SIV-infection, Tfh accumulation is associated with increased frequency of GC B cells and SIV-specific antibodies [15,16]. Interestingly, when cocultured *in vitro* with autologous B cells, Tfh cells from HIV-infected subjects fail to provide adequate B cell help, which may be related to the increased PD-L1 expression on B cells [17]. Tfh cells are also targets of HIV infection, replication and production [13]. Regarding humoral responses, HIV infection impairs B cell maturation causing a loss of memory B cells and an expansion of plasma cells [14,17–19]. It should be noted that most of these studies were restricted to lymph nodes.

Because spleen is the largest lymphoid organ and the place where immune responses against blood-borne pathogens are elicited, we decided to characterize splenic Tfh cell populations of HIV-infected individuals. During the 90s, before introduction of Highly Active Antiretroviral Therapy (HAART), Autran and colleagues collected spleen samples from HIV^-^ donors and untreated HIV-1^+^ individuals [20–24]. At that time, immune thrombocytopenic purpura (ITP) was a frequent complication of HIV infection. To resolve thrombocytopenia, splenectomy was proposed as treatment for HIV^+^ patients who did not respond to standard therapy [25]. Studies of such clinical specimens led to important breakthroughs in the comprehension of HIV interference with immune system. One of the most important work performed on HIV-infected white pulps revealed for the first time, that infected CD4 T cells are present in lymphoid area where they might be eliminated by HIV-specific cytotoxic T lymphocytes infiltrated in the spleen [24]. Indeed, a topological study of the CTL response showed that HIV specific cytotoxic T lymphocytes (CTL) co-localize with HIV-producing cells in germinal centers [26], suggesting an efficient CD8 priming *in vivo*. In line with this, antiviral CTL immune responses directed against Nef protein have been evidenced [27]. Taking advantage of this unique collection of splenocytes, we performed an extensive characterization of splenic Tfh and B cells from HIV^+^ and HIV^-^ individuals.

Here, we uncover that Tfh, GCTfh and Tfr cells from the spleen of HIV-infected patients are expanded as compared to those of uninfected individuals. However, chronic HIV-infection severely impacts the transcription profiles of splenic Tfh and GCTfh, particularly affecting the expression of genes implicated in costimulation, inhibition and signal transductions. We show that splenocytes from HIV^+^ individuals fail to produce IL-10 and IL-4 that are of particular importance for B cell maturation and the regulation of GC reaction. As a putative consequence of Tfh and Tfr dysfunctions, we evidenced a skewed B cell maturation towards an expansion of GC B cells population at the cost of memory B cells in HIV^+^ spleens.

## Materials and Methods

### Samples

Spleens were obtained from organ transplant donors or from patients requiring therapeutic splenectomy. The spleen collection consists of 4 groups: HIV negative Immune Thrombocytopenic Purpura negative (HIV^-^ITP^-^) (n = 9), HIV^+^ITP^-^ (n = 8), HIV^-^ITP^+^ (n = 4), HIV^+^ITP^+^ (n = 10). All spleen samples included in this study were collected following national ethical guidelines regulating the use of human tissues and have already been described in previous studies: HIV^-^ITP^-^ [28], HIV^+^ITP^-^ [29,30], HIV^-^ITP^+^ [31], HIV^+^ITP^+^ [23,26]. Note that HIV+ spleens were isolated from individuals classified in chronic stage (from middle to late stage) of HIV infection and who did not receive any HAART [31].

### Ethics statement

All samples included in this study were collected following national ethical guidelines regulating the use of human tissues. All adult subjects provided informed consent, and a parent or guardian of any child participant provided written informed consent on their behalf. Patient samples were collected according to French Ethical rules. Written informed consent and approval by Institutional Review Board at the Pitié-Salpêtrière Hospital were obtained for this study.

### Splenocytes stimulation

Splenocytes from HIV^-^ITP^-^ (n = 4), HIV^+^ITP^-^ (n = 4), HIV^-^ITP^+^ (n = 3), HIV^+^ITP^+^ (n = 5) were cultured at 37°C in 6 well plates at a concentration of 1 X 10^6^ cells/mL in complete RPMI medium containing 10% FBS containing IL-2 (0.2U/mL), Penicillin/streptomycin (Gibco) and when stated were activated for two days using PHA (PAA) (1μg/mL).

IL-10, IL-6, IL-1ß, IL-4 concentrations were evaluated in supernatants from PHA stimulated splenocytes by Cytometric Beads Array high sensitivity assay according to the manufacturer protocol (BD Biosciences).

### Flow cytometry

#### Antibodies

Directly conjugated antibodies were purchased from BD Biosciences (San Jose, CA, USA): CCR7-PECy7 (3D12), CD3-PerCPCy5.5 and CD3-V500 (UCHT1), Foxp3-AF488 (259D/C7), CD25-AF700 (M-A251), CD4-APCH7 (RPA-T4), CXCR5-AF647 (RF8.B2), CD45RA-PECF594 (HI100), CD19-APCCy7 (SJ25C1), IgM-FITC (G20-127), IgD-PECF594 (IA6-2), CD38-V450 (HB7), CD138-PerCPCy5.5 (MI15), CD21-APC (B-LY4), CD27-PE (M-T271), IgG-BV605 (G18-145) or BioLegend (San Diego, CA, USA): PD1-PE (EH12.2H7), CD19-BV650 (HIB19), CD57-PB (HCD57), ICOS-FITC and ICOS-PerCPCy5.5 (C398.4A), OX40-PECy7 (BER-ACT35), CD40L-BV605 (24–31).

#### Membrane staining

Cells were washed twice with PBS1X containing 0.5% BSA and 2mM EDTA, pre-incubated 5 minutes at room temperature with anti-CXCR5 and anti-CCR7 antibodies and then stained for 20 minutes at room temperature with the corresponding mix of conjugated antibodies.

#### Intracellular Foxp3 staining

Cells were washed and permeabilized using Human Foxp3 Buffer Set (BD Biosciences) according to the manufacturer’s recommendation and stained for 30 minutes at room temperature with antibody Foxp3-AF488 (259D/C7) from BD Biosciences.

Cell acquisition was performed with a Fortessa cytometer (BD Biosciences) and data was analyzed with BD FACSDiva software (BD Biosciences) or FlowJo (Tree Star, Ashland, OR, USA).

### Assessment of integrated proviral DNA using qRT-PCR

A fraction of the naive, memory, Tfh and GCTfh CD4 cells sorted from infected spleens were reserved and conserved at -80°C in RNA later (Qiagen). Proviral integration was assessed by quantitative real-time PCR (qRT-PCR) for HIV-gag DNA, as previously described [32].

### Fluidigm Gene Expression analysis

#### Cell sorting and reverse transcription

Splenocytes from 5 HIV^+^ITP^+^ and 4 HIV^-^ITP^-^ patients were thawed and stained with the “Tfh sorting” panel (**S2 Fig**).

One hundred cells of each population were collected by FACS sorting using a FACSAria II (BD Biosciences) in a RT-PreAmplification Reaction mix containing the pooled TaqMan primers (0.2X of each) and the SuperScriptIII Platinium Taq Polymerase from the CellDirect OneStep qRT-PCR Kit (Invitrogen). Reverse transcription and gene-specific pre-amplification were performed (18 cycles). cDNA was diluted 1/5 in DNA suspension buffer (Teknova), and stored at 4°C.

#### Fluidigm dynamic array

Samples and assays (primer pairs) were prepared for 96.96 Fluidigm Dynamic arrays (Fluidigm, San Fransisco, CA, USA) according to the manufacturer’s recommendation. Briefly, assays were prepared by dispensing 20X Fluidigm Gene Expression assay Loading Reagent and the matching 20X TaqMan Gene Expression Assay into the wells of a 10X Assay Plate. Samples were mixed with 20X Fluidigm Sample Loading Reagent and TaqMan Universal PCR Master Mix 2X (Applied Biosystems). A 96.96 Dynamic Array primed chip was then loaded with assays and samples, and the real time-PCR was run on a Biomark System for Genetic Analysis according to the Fluidigm Protocol. All reactions were made in duplicate. Data were collected and analyzed using Fluidigm Real-Time PCR Analysis software.

#### Multivariate analysis

Multivariate analysis allows for the simultaneous consideration of all investigated genes, for the identification of genes that contributed most to the total variance between HIV+ and control samples. SAS-JMP software version 10.0.0 (SAS Institute Inc., Cary, NC, USA) was used for multivariate analysis: Principal component Analysis and unsupervised hierarchical clustering (**S3 Fig**).

### Statistics

Statistical significance (p-values) was obtained using the non parametric Mann-Whitney U test. *p<0.05, **p<0.005, ***p<0.001. Error bars depict mean +/- standard error of the mean (SEM) in all bar graphs shown. The GraphPad Prism statistical analysis program (Graph-Pad Software, version 5.0) was used throughout.

## Results

### Impact of chronic HIV infection on CD4 T and B cell distribution in the spleen

Using flow cytometry, we evaluated the impact of HIV infection on the distribution of CD4 T cell subsets and B cell maturation in splenocytes. Spleen samples consisted of 4 groups including HIV negative ITP negative (HIV^-^ITP^-^) (n = 7), HIV^-^ITP^+^ (n = 3), HIV^+^ITP^-^ (n = 4), HIV^+^ITP^+^ donors (n = 8). All HIV^+^ spleens came from individuals classified in the chronic stage (from middle to late stage) of HIV infection who did not receive HAART.

**Fig 1:**
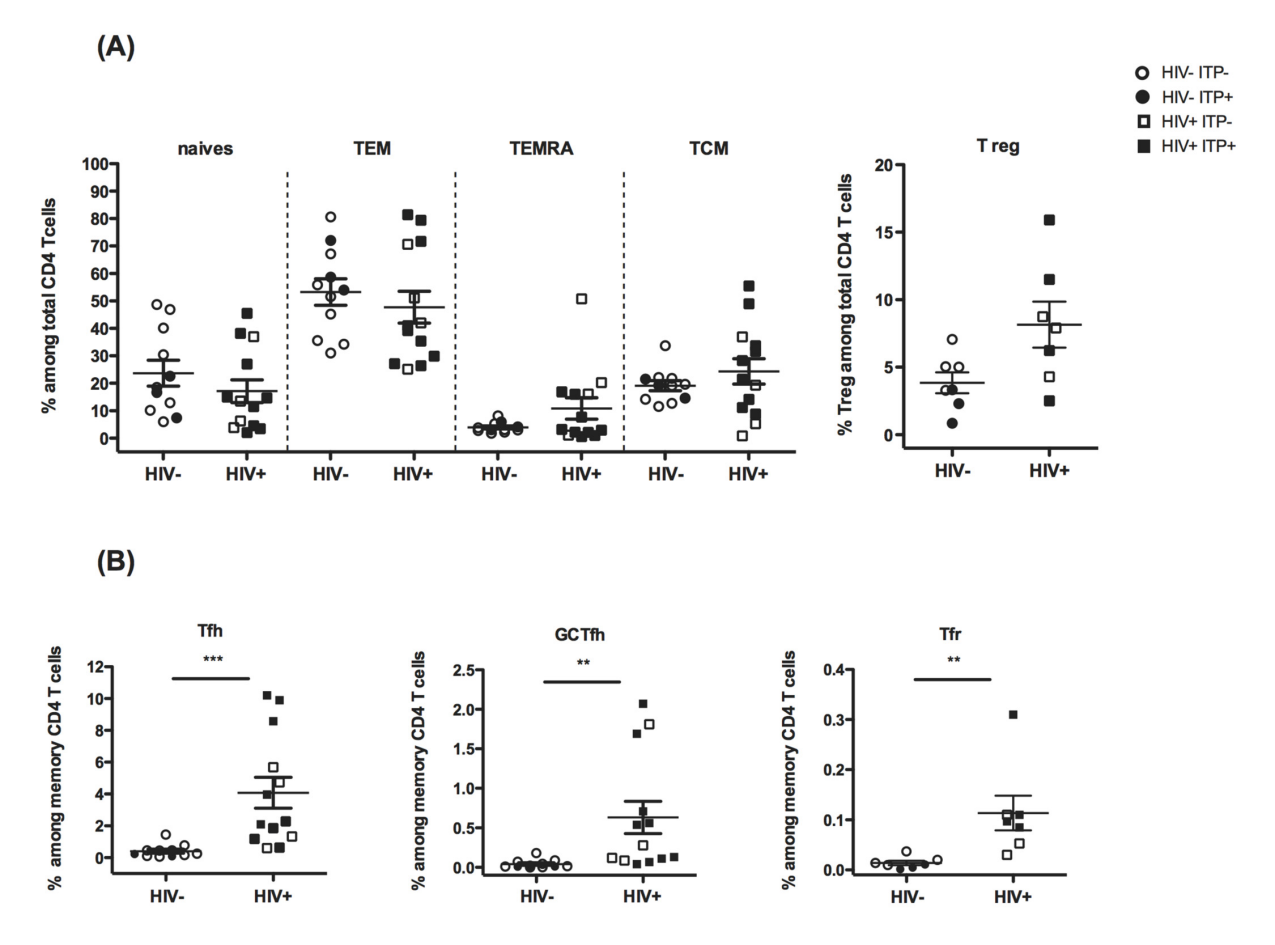
Tfh cell subsets, including Tfr, are present in greater proportions in HIV^+^ spleens. (A) Splenocytes were stained for T helper cell markers and analyzed by flow cytometry: according to the expression of CD45RA and CCR7, CD4 T cells were subdivided into CD45RA^+^CCR7^+^ naïve, CD45RA^-^CCR7^-^ effector memory (TEM), CD45RA^+^CCR7^-^ terminally differentiated effector memory (TEMRA) and CD45RA^-^CCR7^+^ central memory (TCM) T cells. Regulatory T (Treg) cells were identified as CD4^+^ CD45RA^-^ Foxp3^+^ CD25^+^. HIV^-^ITP^-^ n = 8, HIV^+^ITP^-^ n = 4, HIV^-^ITP^+^ n = 3 and HIV^+^ITP^+^ n = 9. (B) Tfh cells were identified as CD3^+^ CD4^+^ CD45RA^-^ CCR7^-^ CXCR5^+^, GCTfh are the CD57^+^ subset of Tfh, and Tfr are Foxp3^+^ Tfh cells (**S1 Fig**). The frequency of Tfh, GCTfh and Tfr cells is represented as the percentage of total memory CD4^+^ T cells. HIV^-^ITP^-^ n = 8, HIV^+^ITP^-^ n = 4, HIV^-^ITP^+^ n = 3 and HIV^+^ITP^+^ n = 9. Frequency of Treg and Foxp3^+^ Tfh (Tfr) were identified for HIV^-^ITP^-^ n = 4, HIV^-^ITP^+^ n = 3, HIV^+^ITP^-^ n = 3 and HIV^+^ITP^+^ n = 4. Statistics were obtained using the non parametric Mann-Whitney test *p<0.05, **p<0.005, ***p<0.001.

First, we investigated the distribution of CD4 T cells among naïve, central memory (TCM), effector memory (TEM), terminally differentiated effector memory cells with reacquired CD45RA (the marker of naïve cells) (TEMRA) and regulatory T cell (Treg) (**Fig 1A**). Proportions of naïve, TCM TEM and TEMRA CD4 T cell subsets were not affected by HIV infection. Interestingly, HIV^+^ spleens exhibited a slight increase of the Treg population, while this population was decreased (p<0.05) in HIV^-^ITP^+^ spleens, as compared to HIV^-^ ITP^-^ spleens (**Fig 1A**).

We then focused on the follicular helper T cell populations (**Fig 1B and S1 Fig**). We identified splenic Tfh cells as memory T helper cell expressing CXCR5^high^ PD-1^high^ but not CCR7. CD57 expression was used to characterize GCTfh [33] (**S1 Fig)**. We observed that the percentages of Tfh and GCTfh cells were significantly increased among memory CD4 T cells **(Fig 1B)**. Overall, this increase is strictly restricted to HIV+ individuals independently of their ITP status. HIV^+^ITP^-^ samples exhibit a significant increase of Tfh cell (p = 0.0141) and of the GCTFh cell (p = 0.0131) frequencies, as compared to HIV^-^ITP^-^, whereas we observed no significant differences between HIV^-^ITP^-^ and HIV^-^ITP^+^ samples. Of note, Tfh and GCTfh cell frequencies were similar between HIV^+^ITP^-^ and HIV^+^ITP^+^ samples (not shown) confirming that the ITP status does not impact Tfh cells distribution in these samples.

We also analyzed the Tfr cell population, the regulatory counterpart of Tfh cells, within the spleens (**Fig 1B**). Tfr express all the characteristic markers of Tfh, as well as Foxp3 and CD25 markers (**S1 Fig**). Remarkably, the proportion of Tfr among memory CD4 T cells was significantly increased in HIV^+^ splenocytes as compared to HIV^-^ITP^-^ and HIV^-^ITP^+^ individuals **(Fig 1B)**.

**Fig 2:**
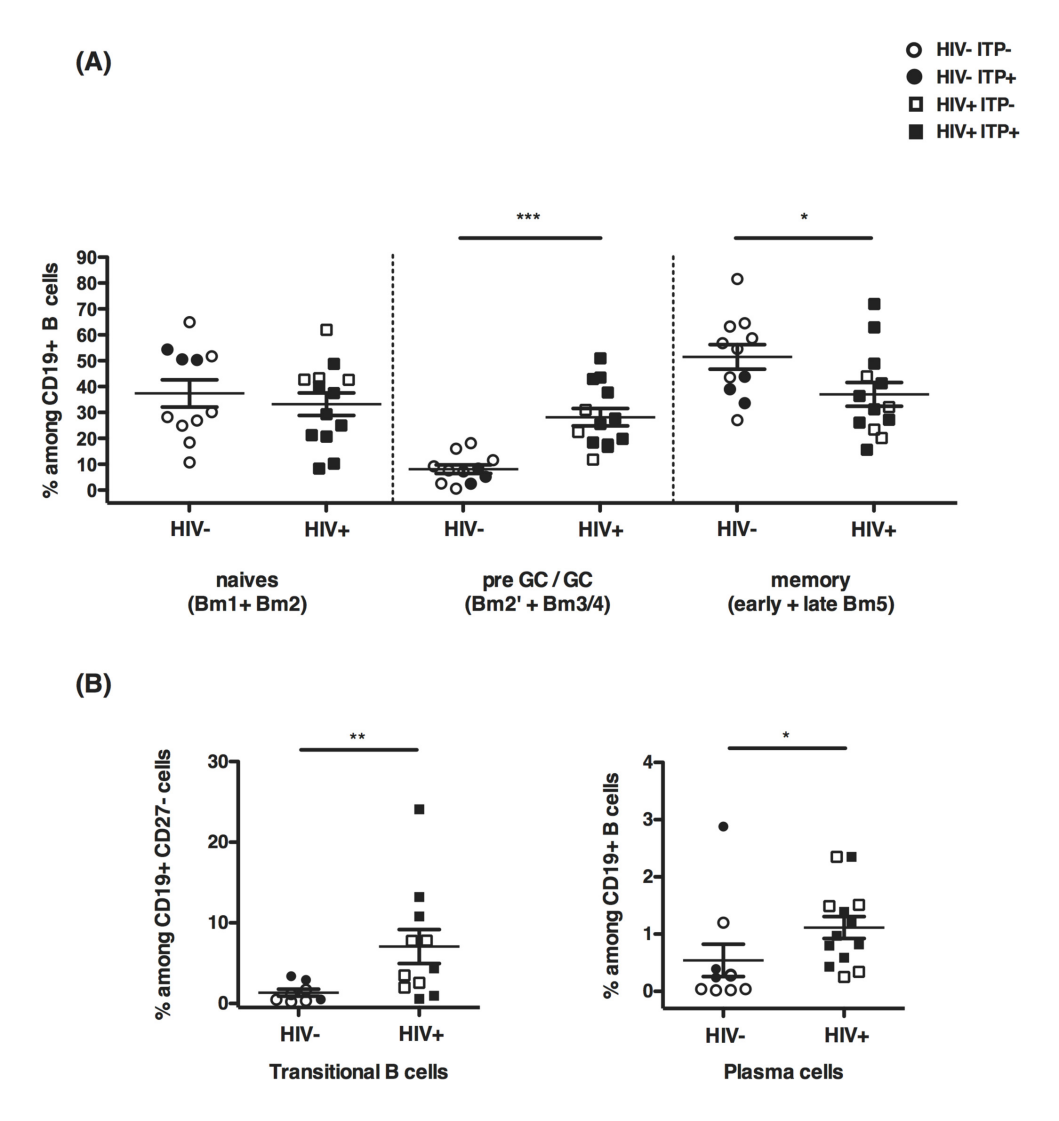
GC B cells accumulate whereas memory B cell compartment is reduced in HIV+ spleens. (A) Using flow cytometry, B cell maturation was assessed according to the expression of CD38 and IgD markers by CD19^+^ cells (34). The gating strategy is presented in **S1 Fig**. In HIV chronically infected patients, B cell maturation is biased towards GC B cells and PC subsets as compared to HIV^-^ donors and ITP^+^ patients. (B) The transitional B cell population was identified as CD19^+^CD38^++^IgD^+^CD27^-^IgM^+^ and plasma cells as CD19^+^CD38^++^IgD^-^. In the box-and-whiskers plot, box size represents the limits of data for the second and third quartiles, with medians shown as bars. Whiskers define the minimum and maximum of the data presented. HIV^-^ITP^-^ n = 8, HIV^-^ITP^+^ n = 3, HIV^+^ITP^-^ n = 5 and HIV^+^ITP^+^ n = 8. Statistics were obtained using the non parametric Mann-Whitney test *p<0.05, **p<0.005, ***p<0.001.

We then asked whether Tfh and Tfr cell expansions might be associated, in the spleens, with alterations of the B cell compartment. To this end we used flow cytometry, to characterize the different stages of differentiation from naïve B cells to memory B cells and antibody-secreting plasma cells [34] (**S1 Fig**). The percentage of naive B cells, defined as Bm1 and Bm2 populations among total B cells (**S1 Fig**), was comparable in HIV-infected spleens as compared with uninfected (**Fig 2A**). In contrast, the percentages of the pre-GC and GC B cells, defined as Bm2’ and Bm3/4 respectively (**S1 Fig**) were significantly increased in both HIV^+^ITP^-^ and HIV^+^ITP^+^ samples (**Fig 2A**). In addition, in the context of HIV-infected spleens, the ratio of memory B cells, defined as early Bm5 and late Bm5 was decreased, independently of the ITP status (**Fig 2A**). Remarkably, transitional B cells (CD19^+^CD38^++^IgD^+^CD27^-^IgM^+^) were significantly increased in HIV^+^ spleens as compared to HIV^-^ITP^-^ and HIV^-^ITP^+^ samples (**Fig 2B**). Plasma cells (CD19^+^CD38^++^IgD^-^) were also slightly increased in HIV^+^ spleens (**Fig 2B**).

Altogether our data unravel that Tfh cell subsets, including Tfr, are present in greater proportions in HIV^+^ spleens. Pre-GC and GC B cells also accumulate in the spleen while further differentiation is rerouted to plasma cells at the cost of memory B cell differentiation as already reported in lymph nodes by Lindqvist et al. [14].

### Transcriptional profiles of splenic Tfh and GCTfh cells

Recent studies performed with the blood or lymph nodes of HIV-infected donors, suggested that Tfh cells might be functionally altered as compared to Tfh from uninfected individuals [13,14]. We therefore investigated some functional attributes of Tfh from the spleen. To this end, we performed a comparative transcription profile analysis between Tfh and GCTfh from chronically HIV^+^ and HIV^-^ spleens. Expansion of splenic Tfh and GCTfh cells is restricted to HIV^+^ individuals independently of their ITP status (**Fig 1B**). Thereafter and because of the scarcity of spleen samples from HIV^+^ITP^-^ donors, we performed the transcriptome analysis on HIV+ITP+ samples. GCTfh and Tfh were isolated by cell sorting based on the expression of CXCR5 and PD-1 markers. GCTfh cells express the CD57 marker that allows their identification. **S2 Fig** shows the gating strategy used to sort splenic Tfh and GCTfh from 4 HIV^-^ITP^-^ (A-D) and 5 HIV^+^ITP^+^ (F-J) donors. Sorted cells were used to perform high throughput gene expression measurement with real time PCR in a microfluidic dynamic array. Using this approach, we quantified the expression of 96 genes of interest in 100 cells. After cleansing and normalization, consistent data were used for statistical analysis. We focused on genes specifically implicated in T cell functions (**S1 Table**).

**Fig 3:**
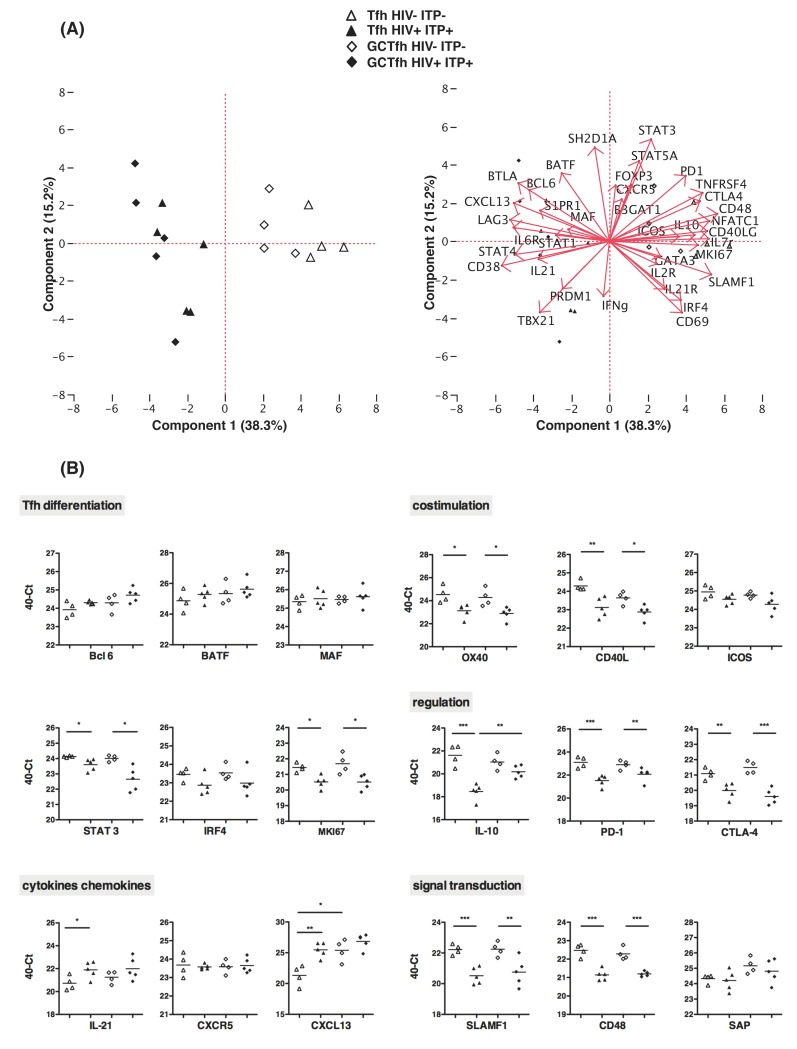
HIV infection severely impacts transcription profile of splenic Tfh and GCTfh cells. GCTfh and Tfh were sorted using flow cytometry based on the expression of CXCR5 and PD-1 associated or not with CD57 marker respectively (**S2 Fig**). Sorted cells were used to perform high throughput gene expression measurement with real time PCR in a microfluidic dynamic array. After cleansing and normalization, consistent data were used for statistical analysis. We focused on genes specifically implicated in T cell functions (**S1 Table**). (A) 2D Principal component analysis (PCA) plot representation of gene expression profile of Tfh (triangle) and GCTfh (diamond) cells from HIV^-^ ITP^-^ (n = 4, empty symbols) and HIV^+^ ITP^+^ (n = 5, filled symbols) spleens. The projection of the data on the first and second principal components efficiently discriminates HIV^+^ samples, suggesting that HIV-infection deeply impacts gene expression profile of Tfh and GCTfh cells. (B) mRNA expression in Tfh and GCTfh sorted from splenocytes of uninfected (n = 4) and HIV^+^ (n = 5) individuals. The data are expressed in 40-Ct where Ct represents the threshold cycle number and 40 is chosen because the PCR run stops after 40 cycles. This value is directly correlated with the initial amount of RNA and therefore allows a quantitative comparison for 18 genes encoding cytokines/chemokines and proteins implicated in Tfh differentiation, costimulation, immune regulation and signal transduction. Statistics were obtained using the non parametric Mann-Whitney test *p<0.05, **p<0.005, ***p<0.001.

First, using JMP statistical software, we performed a principal component analysis (PCA) to reduce the multidimensional gene expression data set to lower dimensions for analysis, by retaining genes that contributed most to the total variance (**Fig 3A**). The projection of the data on the first component (38.3% variance explained) efficiently discriminated HIV^+^ samples, suggesting that HIV infection deeply impacts gene expression profile of Tfh and GCTfh cells (**Fig 3A, Top panel**). Particularly, component 1 revealed a bias of Tfh from HIV^+^ spleens to preferentially express genes implicated into Tfh cell differentiation and Th1 functions whereas Tfh from HIV^-^ spleens more highly expressed genes implicated in costimulation, inhibition and signal transduction functions (**Fig 3A, lower panel and S1 Table**).

Genes displaying significant differences in their expression levels between Tfh cells from HIV^+^ and HIV^-^ donors are presented in **Fig 3B**. Gene expression levels between Tfh and GCTfh cells were similar except in two cases: in HIV^-^ samples, *CXCL13* gene expression was significantly enhanced in GCTfh samples as compared to Tfh and in HIV^+^ samples, *IL-10* was greatly impaired in Tfh as compared to GCTfh. Remarkably, the expression levels of genes implicated in Tfh differentiation such as *BCL6*, *BATF*, and *MAF* were not affected by HIV infection, appearing slightly increased in Tfh and GCTfh from HIV^+^ samples **(Fig 3B)**, while *IL-21* and *CXCL13* expression, two key mediators of Tfh function, were increased in Tfh from HIV^+^ spleens. In contrast, the expression level of signal transducer and activator of transcription (*STAT*)-3 gene, which is required for full Tfh differentiation [35] was significantly reduced in Tfh and GCTfh from HIV^+^ spleens as compared to HIV^-^ samples. In addition, HIV infection significantly compromised the expression of the costimulatory molecules OX40 (TNFRSF4), CD40L and, to a lesser extent, ICOS encoding genes, in both Tfh and GCTfh. Very interestingly, the expression of genes encoding immune regulatory molecules such as IL-10, CTLA4 and PD-1 was also severely impacted by HIV infection in both Tfh and in GCTfh. Of note, the expression of the proliferation marker Ki67 mRNA was also notably reduced. Finally, our results indicated that gene encoding SLAMF1 and CD48 receptors that belong to the family of the Signaling lymphocytic activation molecule (SLAM) are significantly impacted by HIV infection. Of note, SLAMF1 is specifically required for IL-4 production by GCTfh [36]. In contrast, the expression of SLAM-associated protein (SAP), known to play a critical role in the sustained adhesion between T and cognate B cell [37,38] is not compromised by HIV-infection. In conclusion in HIV+ spleens, the expression of most genes implicated in Tfh and GCTfh differentiation was not compromised with the notable exception of *STAT-3* suggesting that only the final stage of Tfh differentiation might be affected by HIV-infection. In contrast, the expression of genes implicated in Tfh function, such as costimulation, immune regulation or signal transduction were severely reduced in HIV-infected spleens.

Unsupervised hierarchical clustering grouped together GCTfh from HIV^+^ samples (**S3 Fig**). Hence, *CXCL13*, *IL-21*, and *CXCR5* genes were highly expressed in GCTfh cells from HIV^+^ samples as well as those encoding transcription factors implicated in Tfh differentiation (*MAF*, *BATF*, *Bcl6*). These observations might explain the higher proportion of GCTfh in HIV^+^ spleens.

### Ex-vivo cytokine production by splenocytes

To evaluate whether our observations on the skewed transcription profiles of Tfh cells might modulate the splenic microenvironment we analyzed *ex vivo* the capacity of activated splenocytes to secrete various cytokines. This question could not be addressed directly using Tfh cells since their low frequency in HIV^-^ spleens did not allow their sorting and assessement of functional properties such as cytokine secretions. Thereafter, cytokine secretion profiles were evaluated using whole splenocytes.

**Fig 4:**
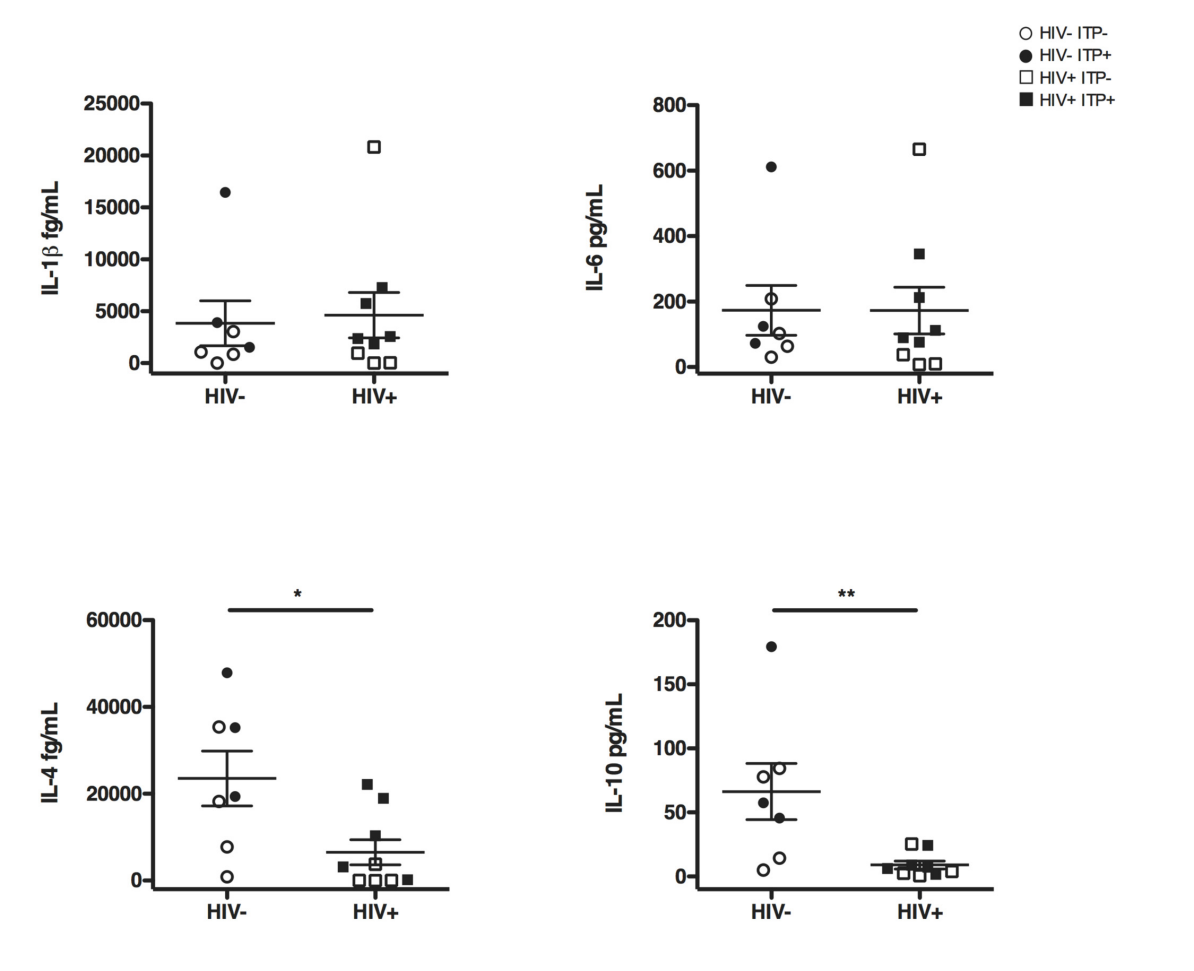
Activated HIV+ splenocytes fail to produce IL-4 and IL-10: Total splenocytes were stimulated with PHA for 2 days and culture supernatants were analyzed for IL-1ß, IL-6, Il-4 and IL-10 using enhanced sensitivity BD CBA flex set assay. Horizontal bars represent mean and error bars show SEM. HIV^-^ITP^-^ n = 4, HIV^-^ITP^+^ n = 3, HIV^+^ITP^-^ n = 4 and HIV^+^ITP^+^ n = 5. Statistics were obtained using the non parametric Mann-Whitney test *p<0.05, **p<0.005, ***p<0.001.

To this end, splenocytes from HIV^-^ITP^-^ (n = 4), HIV^-^ITP^+^ (n = 3), HIV^+^ITP^-^ (n = 4) and HIV^+^ITP^+^ (n = 5) were activated using PHA and secretion of IL-10, IL-4, Il-6 and IL-1ß were quantified (**Fig 4**).

We observed a profound defect of HIV^+^ splenocytes to produce IL-10 and IL-4, two major cytokines produced by Tfh cells and related to B cell maturation. This observation is restricted to HIV^+^ samples independently of the ITP status since no significant difference was observed when comparing ITP^-^ and ITP^+^ samples (not shown). Strikingly, IL-6 and IL-1ß productions were not affected by HIV-infection (**Fig 4**). IL-6 secretion is classically linked to viral infection. However Shive et al. have demonstrated that IL-6 secretion is not correlated with HIV-RNA. Moreover, histoculture of HIV chronically infected lymph nodes as well as histoculture of HIV^-^ lymph nodes *in vitro* infected with HIV do not reveal any difference in the amount of IL-6 secretion as compared to control lymph nodes [39].

Thus, our results demonstrated a defective IL-10 and IL-4 production by activated splenocytes and provided new evidence to explain both defective B cell differentiation and Tfh cells abundance observed in HIV^+^ individuals.

### HIV-1 DNA integration in splenic Tfh cells

**Fig 5:**
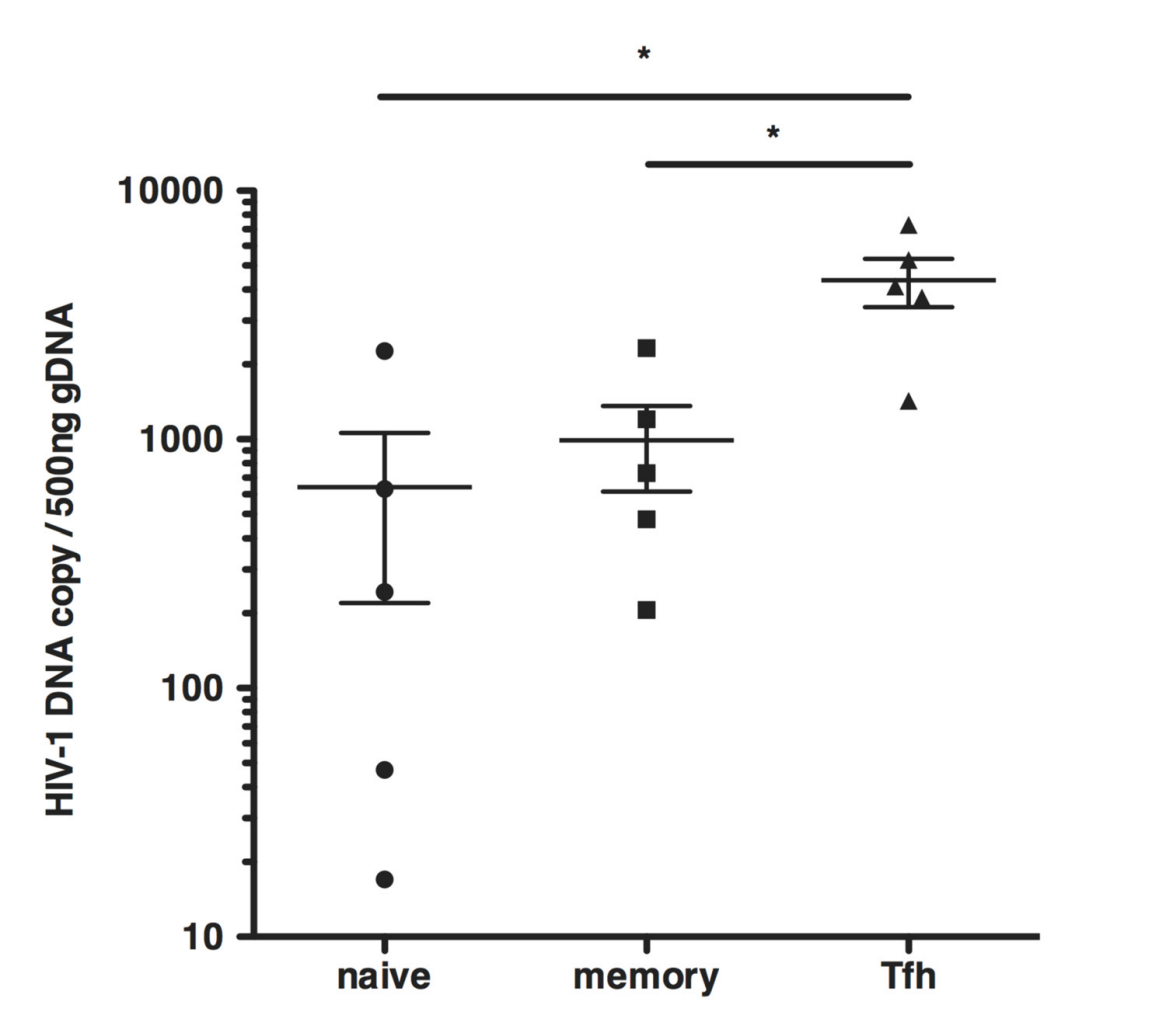
Splenic Tfh subset harbor high amount of HIV-1 DNA integration. qRT-PCR quantification of HIV proviral DNA levels in GCTfh, Tfh, memory and naïve CD4^+^ T cell subsets isolated from splenocytes of five HIV^+^ ITP^+^ individuals as described in **S1 Fig**. Symbols represent individual samples: horizontal bars represent mean; and error bars show SEM. Statistics were obtained using the non parametric Mann-Whitney test *p<0.05, **p<0.005, ***p<0.001.

CD4+ T cells being the major target for HIV infection, we then asked whether HIV might infect splenic Tfh cells. To address this question, we sorted CD3^+^CD4^+^CD45RA^+^ naïve, CD3^+^CD4^+^CD45RA^-^ICOS^-^ resting memory, and CD3^+^CD4^+^CD45RA^-^ICOS^+^PD-1^high^CXCR5^+^ total Tfh CD4 T cell populations from chronically HIV^+^ subjects (HIV^+^ITP^+^ n = 5) and quantified integrated HIV proviral genomes (**Fig 5**). HIV proviral DNA copy numbers were normalized to genomic DNA input (**Fig 5**).

Our results indicated that whereas resting memory CD4 T cell populations harbored only a slightly higher amount of HIV-DNA copies than naïve CD4T cells, the Tfh subset harbored a significantly higher amount of HIV DNA (more than 4-fold increase). Our data obtained using HIV-infected spleens are in accordance with observations using lymph nodes **[13].** This raises an apparent paradox between the elevated proportions of Tfh cells population and their activated status and their higher susceptibility to HIV integration.

## Discussion

The recent discovery of broad and potent HIV-1 neutralizing antibodies (bNAbs) has renewed optimism for developing an effective vaccine against HIV-1 [40]. The generation of bNAbs requires multiple rounds of B cell receptor (BcR) affinity maturation, suggesting a crucial role of Tfh in their generation [41]. However, less than 1% of HIV-infected patients develop bNAbs that arise late in the infection, 2 to 4 years after infection, indicating probable Tfh and B cell dysfunctions in HIV infection [42].

Until now, most studies have investigated human Tfh subsets in lymph nodes of HIV-infected patients [13,14,17]. However, spleen constitutes another major compartment for the induction of adaptive immunity against blood borne pathogens such as HIV. Here, using a unique collection of cryopreserved spleens cells from HIV-infected individuals, we performed an extensive characterization of Tfh, GCTfh and Tfr cells. Similar to what has been already reported in lymph nodes, the percentages of Tfh and GCTfh cells were significantly increased in HIV^+^ spleens, while ITP status did not impact these populations. We also reported a slight increased Foxp3^+^ Treg population in accordance with reports of highly elevated Treg levels in mucosa of untreated chronically HIV^+^ patients [43]. For the first time we showed that Tfr proportions were significantly increased in HIV^+^ spleens. These results suggest that the expansion of Tfh and GCTfh populations might not result from a scarcity in Tfr cells, as suggested in HIV^-^ITP^+^ spleen samples studies [44]. The question of Tfr suppressive functions would be very interesting to address in those samples. However due to the lack of specific cell surface markers identifying Tfr, splenic Tfr cells could not be sorted and tested for their suppressive function on Tfh proliferation. Moreover, since the development of Tfr cells kinetically follows Tfh amplification in order to regulate the GC reaction [10] one can hypothesize that the GC reaction is sustained but not completed in chronically HIV^+^ spleens.

HIV infection impairs B cell maturation causing a loss of memory B cell and an expansion of plasma cells [13,14,18,19,45]. Of note, loss of memory B cells during HIV infection is also due to apoptosis [46]. Here, we reported a skewed B cell distribution in HIV^+^ spleens: pre-GC and GC B cells accumulated while further differentiation was rerouted to plasma cells at the cost of memory B cell differentiation. The loss of memory B cells has already been noted in the spleens from SIV-infected macaques [47]. Hence, although increased in HIV^+^ spleens, Tfh and GCTfh populations were not able to support complete B cell differentiation from naïve to memory phenotype. Remarkably, excessive numbers of Tfh cells can result in B cells losing their affinity for antigen or even becoming self-reactive [48]. In line with this, it has been shown that splenic Tfh expansion participates in anti-platelet-antibody production in ITP [49]. In our collection of HIV^-^ ITP^+^ samples, we did not observe enrichment in Tfh cells as compared to HIV^-^ ITP^-^, further highlighting that the increase of Tfh cells in HIV^+^ samples is most likely a consequence of HIV-infection. Many autoimmune disorders, such as ITP, have been shown to have a higher incidence among HIV^+^ individuals [50]. Of note, a recent study reported the characterization of an anti ds-DNA antibody that also broadly neutralizes HIV in a SLE/HIV individual [51].

Perreau et al. have already shown that Tfh cells serve as a major CD4 T cell compartment for HIV infection, replication and production in lymph nodes from viremic HIV^+^ patients [13]. Consistent with their data, we reported a higher amount of HIV-1 DNA integration in total splenic Tfh cell populations than in naïve and resting memory CD4 T cells. However, according to the phenotypic characterization of *ex vivo* splenic CD4 T cell populations, Tfh cells were in higher proportion in HIV^+^ individuals. This raises an apparent contradiction between the elevated proportions of Tfh cells and their higher susceptibility to HIV infection. One possible explanation is that antigen persistence could drive CD4 T cells away from antiviral Th1 toward Tfh differentiation [52]. Hence, one can hypothesize that untreated patients in chronic stage of HIV infection present high amounts of viral antigens leading to an exacerbated Tfh differentiation. Interestingly, HIV particles are associated to GC follicular dendritic cells in tonsils and lymph nodes from infected patients [53–55] and Cheynier et al. reported persistence of high level of HIV particles in GC of HIV^+^ spleens from untreated subjects [24]. Thus, antigen persistence combined with immune activation could explain Tfh (and Tfr) expansion despite their higher susceptibility to HIV-integration. In contrast to what reported by Perreau et al. [13], we found that Ki67 mRNA expression was significantly lower in Tfh and GCTfh from HIV^+^ samples than in HIV^-^ samples. However, in accordance with our results, Petrovas et al. reported that higher Ki67 expression by Tfh cells is restricted to the acute phase and is not observed at the chronic stage of SIV infection [15].

This suggests that the higher proportion of Tfh cells was most likely due to a bias towards Tfh differentiation rather than to higher proliferative capacities. Another, non exclusive explanation is that Tfh cells may display restriction factors that are specific or more abundant than in other CD4+ T cells, allowing persistent HIV infection and integration with low proliferation.

The cytokine / chemokine environment might also promote Tfh cells differentiation and their homing into GC [1]. In this study, we showed that expression of IL-21 and CXCL13 encoding genes was enhanced in Tfh from HIV^+^ samples, suggesting that Tfh cells differentiating during HIV infection can contribute to a suitable signaling milieu for GC development.

The key role of Tfh cells is to provide strong B cell helper signals and therefore promote their differentiation into memory B cell displaying high affinity for pathogens. This signal consists of cytokines such as IL-4 and IL-21 and of cell surface molecules such as ICOS and CD40L [1]. Despite the large proportion of follicular T cell subsets in HIV^+^ spleens, gene expression profiling suggested that both Tfh and GCTfh cells were compromised in their capacity to provide strong co-stimulatory signals. CD40L down modulation as been described in total CD4T cell population in late stages of HIV infection [56]. Our transcriptome data using Tfh are in accordance with these observations.

The expression of SLAM molecules was also severely impacted. Combined together, these defects will impact the long lasting T cell / B cell cognate interaction required for the full B cell differentiation into long-lived plasma cells. Very interestingly, SLAMF1 is specifically required for IL-4 production by GCTfh [36], which underlines its major role in GC homeostasis. Hence, our data indicated a defect in *SLAMF1* gene expression that might explain the low level of IL-4 production observed in those spleens. Since IL-4 is one of the major cytokines secreted by Tfh [57] and is required for optimal humoral responses, low IL-4 production displayed by HIV^+^ spleens might contribute to defective GC reaction.

As suggested by our gene expression analysis, IL-10 production was severely compromised in HIV^+^ spleens. Considering that IL-10 is a potent B cell differentiation factor, low level of IL-10 production might contribute to the defective B cell maturation into memory B cells in HIV^+^ spleens. Moreover, IL-10 is a key component of immune regulation in addition to PD-1 and CTLA-4. Here we identified an impaired expression of these inhibitory receptors in the total Tfh population. Interestingly, in addition to PD-1/PD-L1 interactions [17], recent studies reported the major role of the coinhibitory CTLA-4 receptor in the control of the GC reaction by modulating Tfh, Tfr and Treg [58–60]. Therefore, defective expression of IL-10, PD-1 and CTLA-4 might also lead to uncontrolled GC reaction and support Tfh and GCTfh cell differentiation observed in HIV^+^ spleens.

In the context of HIV-infection, the defective STAT-3 encoding gene expression reported here is also of particular interest since it should impact on the full polarization of Tfh. Hence, following acute viral infection, STAT-3 has been reported to repress Type I interferons to promote Tfh cell differentiation, at the cost of Th1 polarization [61]. Therefore, defective STAT-3 expression might explain why Tfh from HIV^+^ spleens harbored a transcription profile biased toward Th1 functions.

Some limitations of the present study should be addressed. First, most of our findings are based on transcriptome profile analysis of sorted Tfh and GCTFh cells and are not confirmed at the level of the protein expression because of the low numbers of cells. Of note, whereas Bcl6 mRNA quantification is not predictive for Bcl6 protein expression [62], STAT-3, CXCR5, CXCL13, IL-4, IL-21, CTLA4 and Il-10 mRNA quantifications have been reported to correlate with the protein levels and most of theses transcripts are biomarkers of pathogenic processes in oncology and transplantation [63–68]. Second, the number of samples is relatively small, notably for HIV^+^ITP^-^ samples. By comparing HIV^-^ITP^-^ with HIV^-^ITP^+^ and HIV^+^ITP^-^ with HIV^+^ITP^+^, we found that ITP status does impact the frequency of Tfh and GCTfh cell populations (not shown). We also verify that IL-4 and IL-10 secretions are not driven by ITP status. These verifications allowed us to combine samples ITP^+^ samples with ITP^-^ in our analysis and reach the sample size that allows statistical significance.

In sum, using unique samples obtained from chronically HIV-infected individuals, we characterized splenic Tfh, GCTfh, and Tfr cells subsets. To our knowledge, this study constitutes the first characterization of Tfh populations, including regulatory cells, in spleen that is the largest lymphoid organ and where the adaptive immune response against blood-borne antigens takes place. We observed that HIV infection leads to a higher proportion of Tfh cells in the spleen as well as a global increase of natural regulatory T and Tfr cells. Remarkably, chronic HIV-infection severely impacted the transcription profile of splenic Tfh and GCTfh, particularly affecting expression of genes implicated in costimulation, inhibition and signal transduction. Moreover, upon activation, the production of IL-4 and IL-10 was severely compromised in HIV^+^ spleens. These multiple failures displayed by Tfh cells and splenocytes from HIV^+^ individuals most likely contributed to impair B cell maturation and differentiation. Indeed in HIV-infected spleens, pre-GC and GC B cells accumulated while further differentiation was re-routed toward plasma cells at the cost of memory B cell differentiation. This skewed B cell differentiation might strongly impact the capacity of the immune system to generate bNAbs. Altogether, our work provides new insights in HIV pathogenesis and explains the global defective humoral responses observed in chronically HIV^+^ patients.

## Supporting Information

S1 Fig Representative flow cytometry contour plots (A) Phenotypic analysis of CXCR5^high^ CCR7^low^ memory CD4 T cells in splenocytes from HIV-infected individuals. Gating strategy used to identify total Tfh, Tfh, GCTfh and Tfr is shown. (B) Representative flow cytometry contour plots showing the B cell maturation for HIV^-^ ITP^-^, HIV^-^ ITP^+^, HIV^+^ ITP^-^ and HIV^+^ ITP^+^ individuals. Bm1 (naïve) CD38-IgD^+^, Bm2 (activated naive) CD38-IgD^++^, Bm2’ (pregerminal center) CD38^++^IgD^+^, Bm3/4 (germinal center) CD38^++^IgD-, early Bm5 CD38+IgD^-^, late Bm5 (CD38-IgD^-^), and plasma cells (PC) CD38^+++^IgD^-^ proportions were determined among CD19^+^ cells.
(TIF)

S2 Fig Gating strategy used to sort Tfh and GCTfh from healthy (n = 4) and HIV^+^ ITP^+^ spleens (n = 5). Sorted cells were used for transcriptome profile analysis (Fluidigm assay).
(TIF)

S3 Fig Unsupervised hierarchical clustering using ward clustering method of 17 selected genes reported to play key roles in Tfh functions: HIV^-^ ITP^-^ (n = 4, A-D) HIV^+^ ITP^+^ (n = 5, F-J). Gene expression has been quantified using the Fluidigm technology in Tfh and GCTfh populations. As compared to healthy controls (green square), Tfh and GCTfh from HIV^+^ samples display a down-modulation of both costimulatory (TNFRSF4, ICOS, CD40LG) and regulatory molecules (CTLA-4, PD-1, IL-10). Expression of genes implicated in signal transduction (*CD48* and *SLAMF1*) is also impaired in HIV+ samples. Tfh and GCTfh cells from HIV^+^ samples highly express genes implicated in Tfh associated functions (*CXCL13*, *IL-21*, *CXCR5*) and genes encoding transcription factors implicated in Tfh differentiation (*MAF*, *BATF*, *Bcl6*) (red square).
(TIF)

S1 Table Name and function of genes associated with T helper function and differentiation. (TIF)
