# Supplementary figures and images for: HIV-Infected Spleens Present Altered Follicular Helper T Cell (Tfh) Subsets and Skewed B Cell Maturation
Source: PLoS One. 2015 Oct 26;10(10):e0140978. doi: 10.1371/journal.pone.0140978 (PMC4621058; doi:10.1371/journal.pone.0140978)

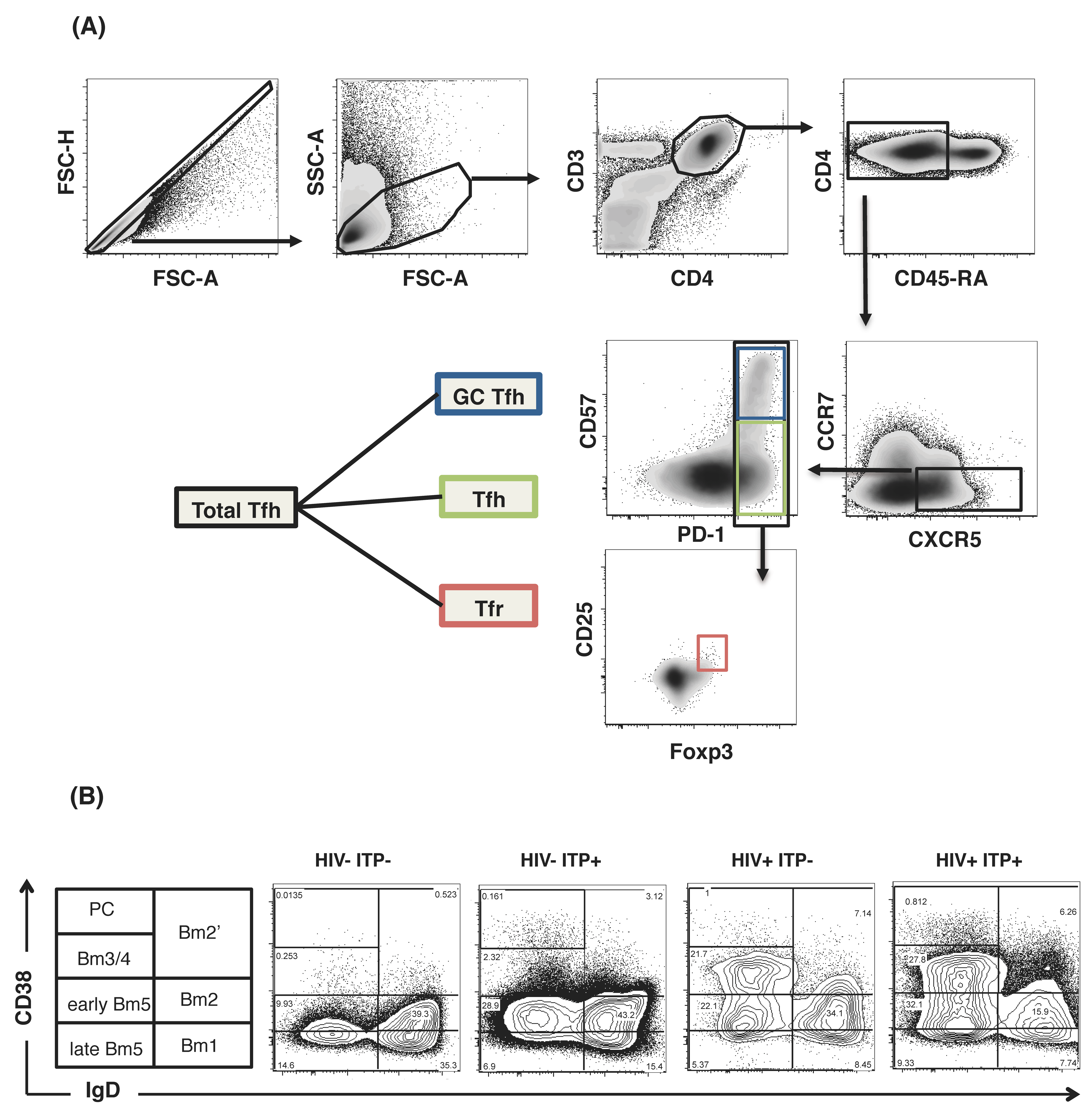

Supplement: S1 Fig — Bm1 (naïve) CD38-IgD+, Bm2 (activated naive) CD38-IgD++, Bm2’ (pregerminal center) CD38++IgD+, Bm3/4 (germinal center) CD38++IgD-, early Bm5 CD38+IgD-, late Bm5 (CD38-IgD-), and plasma cells (PC) CD38+++IgD- proportions were determined among CD19+ cells. (TIF) [file pone.0140978.s001.tif]

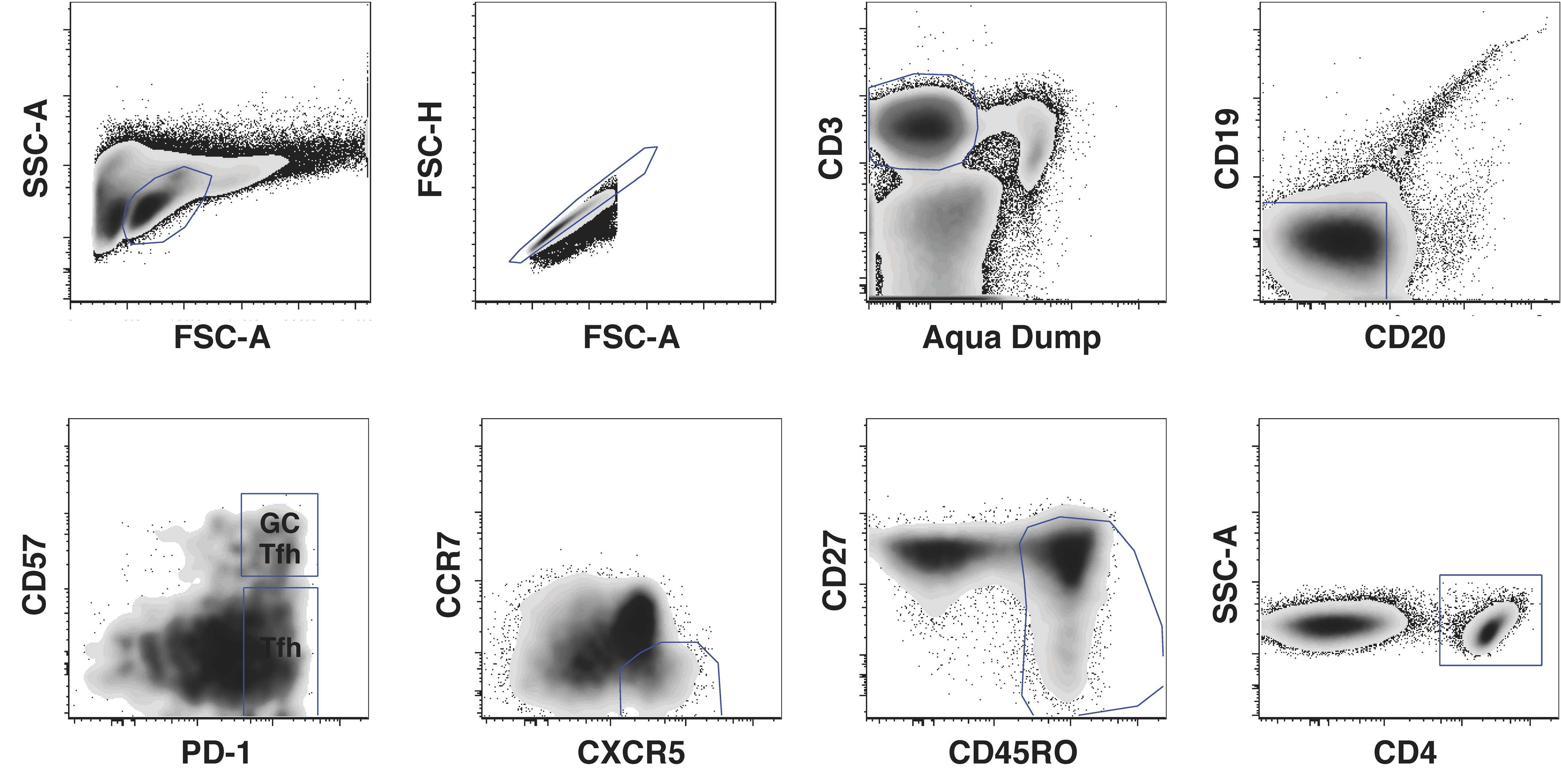

Supplement: S2 Fig — Sorted cells were used for transcriptome profile analysis (Fluidigm assay). (TIF) [file pone.0140978.s002.tif]
